# Harnessing adipose stem cell diversity in regenerative medicine

**DOI:** 10.1063/5.0038101

**Published:** 2021-04-01

**Authors:** Chang Gui, Jacob Parson, Gretchen A. Meyer

**Affiliations:** 1 Department of Biomedical Engineering, Washington University in St. Louis, St. Louis, Missouri 63110, USA; 2 Program in Physical Therapy, Washington University in St. Louis, St. Louis, Missouri 63110, USA; 3 Departments of Neurology and Orthopaedic Surgery, Washington University in St. Louis, St. Louis, Missouri 63110, USA

## Abstract

Since the first isolation of mesenchymal stem cells from lipoaspirate in the early 2000s, adipose tissue has been a darling of regenerative medicine. It is abundant, easy to access, and contains high concentrations of stem cells (ADSCs) exhibiting multipotency, proregenerative paracrine signaling, and immunomodulation—a winning combination for stem cell-based therapeutics. While basic science, preclinical and clinical findings back up the translational potential of ADSCs, the vast majority of these used cells from a single location—subcutaneous abdominal fat. New data highlight incredible diversity in the adipose morphology and function in different anatomical locations or depots. Even in isolation, ADSCs retain a memory of this diversity, suggesting that the optimal adipose source material for ADSC isolation may be application specific. This review discusses our current understanding of the heterogeneity in the adipose organ, how that heterogeneity translates into depot-specific ADSC characteristics, and how atypical ADSC populations might be harnessed for regenerative medicine applications. While our understanding of the breadth of ADSC heterogeneity is still in its infancy, clear trends are emerging for application-specific sourcing to improve regenerative outcomes.

## INTRODUCTION

Since its modern inception, the field of regenerative medicine has dedicated itself to harnessing the power of stem cells. Viewed broadly, the progress is staggering. From organ-in-a-dish models of disease to biomaterial scaffolds to direct stem cell fate *in vivo*, significant progress has been made both in understanding and exploiting the regenerative potential of stem cells. This is especially true of adult stem cells. These cells, which can be derived from the patient's own body and applied without the ethical challenges of embryonic stem cells (ESCs) or the technical hurdles of induced pluripotent stem cells (iPSCs), have pushed a new frontier of stem cell-based therapies. The hematopoietic and mesenchymal stem cells (MSCs) have been the most successful translationally, with over four thousand clinical trials and millions of patients treated (ClinicalTrials.gov). While hematopoietic stem cells are generally restricted to the myeloid and lymphoid lineages, MSCs exhibit plasticity toward a number of adult tissues such as bone, cartilage, adipose, muscle, and even nerve, making them promising candidates for treatment across a spectrum of injury and disease. MSCs are currently under development for the treatment of dozens of conditions from osteoarthritis to infertility.

Mesenchymal stem cells are now recognized to be a highly heterogeneous population. Not only are there variations in proliferative capacity and multipotency between clones of a single isolation^1^ but also well documented differences between donors^2^ and tissue of origin.^3^ Of course, heterogeneity is not all bad. Numerous studies suggest that MSCs from different sources possess preferential plasticity,^4,5^ resilience in specialized environments,^6,7^ and unique immunomodulation,^8,9^ which suggest that the optimal MSC source may be application specific. While there is arguably more heterogeneity between tissue types (e.g., bone marrow vs adipose), there are likely also important differences between anatomical locations within a single type, which have only begun to be explored. Nowhere is this likely to have as big an impact on stem cell-based therapies than in adipose tissue: one because adipose-derived stem cells (ADSCs) show exceptional clinical promise in general and two because adipose tissue has extensive morphological and functional heterogeneity. Nearly all ADSCs in preclinical and clinical studies derive from abdominal subcutaneous fat, but adipose tissue also exists around internal organs, between muscles, and within joints. These other locations or “depots” are thought to be specialized to support adjacent tissues, and each has documented differences in tissue and ADSC function. While typically smaller and less accessible than subcutaneous belly fat, these depots are frequently accessible during surgeries on adjacent tissues and could offer improved regenerative potential as part of an ADSC adjuvant therapy.

In this review, we will discuss the morphological and functional heterogeneity among adipose depots and review the evidence for differential regenerative capacity among their resident ADSC populations. While heterogeneity in adipose depots has long been recognized to influence ADSC regenerative potential, this review moves beyond the traditional abdominal adipose sources to consider specific organ- and tissue-associated depots that could be harnessed to improve regeneration in specialized applications. We will discuss emerging data on the unique properties of ADSCs isolated from these depots and novel strategies to enhance their potential for clinical translation.

## ADIPOSE-DERIVED STEM CELLS: FAT IN A NEW LIGHT

While stem cells have long been the holy grail of regenerative medicine as the body's natural source of regeneration, they are difficult to harness therapeutically. Since the discovery of hematopoietic stem cells in the 1960s and embryonic stem cells (ESCs) in the 1980s, scientists have devoted significant effort to utilize their therapeutic potential in tissue repair and regeneration. Three major issues currently challenge stem cell-based therapies: (1) accessing sufficient quantities of cells, (2) directing those cells to enhance regeneration, and (3) preventing rejection by the body. ESCs have the most regenerative potential in terms of differentiation capacity, but ethical issues concerning their sourcing limit their translational potential. Furthermore, because of their pluripotency and allogenic origin, ESCs have the potential to cause both immune rejection and tumor generation. The potency of ESCs can be regained without the ethical or immune issues in induced pluripotent stem cells (iPSCs) by genetic modification of a patient's own cells, but the expense and low rate of conversion currently limit the translational potential of these cells as well. Adult, or somatic, stem cells avoid the ethical and practical issues with ESCs and iPSCs but have faced other challenges.

Adult mesenchymal stem cells (MSCs) combine multipotency (adipogenic, osteogenic, chondrogenic, myogenic, and neurogenic differentiation capacity) with low immunogenicity (because they can be derived from the patient's own tissues).^10^ Furthermore, they can be easily isolated by enzymatic digestion from nearly every tissue in the body and stably expanded in culture.^11^ The best characterized and most widely used MSCs for cell transplantations are derived from bone marrow. These were the first MSCs isolated for therapeutic use, targeted due to the proregenerative action of bone marrow on fracture healing.^12^ Despite their *in vitro* multipotency and low morbidity during cell culture, the painful harvesting process and relatively low yield limit their potential in clinical applications. Zuk *et al.* were the first to isolate and characterize a similar population of multipotent cells from lipoaspirate.^13^ These adipose-derived stem cells (ADSCs) circumvent some of the challenges associated with bone marrow-derived MSCs because of the abundance and accessibility of adipose tissue. For ADSC isolation, adipose tissue is typically harvested by liposuction, a popular cosmetic surgery yielding hundreds of milliliters of source material. ADSCs are conveniently isolated by enzymatic digestion and filtration and are typically defined as the plastic-adherent population remaining after passaging but may also be sorted by a panel of surface markers (CD31−, CD34+, CD45−, CD90+, CD105+, and CD146−).^14^ With yields as high as 400 000 cells per milliliter, ADSCs require minimal to no culture expansion for many applications.

Application of ADSCs in preclinical models of disease has shown significant regenerative benefits including improvements in cardiac ejection fraction post-infarction,^15^ immunosuppression in rheumatoid arthritis,^16^ learning and memory in Alzheimer's disease,^17^ dystrophin expression in Duchenne muscular dystrophy,^18^ and fibrosis in kidney injury.^19^ Because of their preclinical promise, extensive ADSC-based clinical trials for tissue regeneration and reconstruction are under way to assess their safety and efficiency. The number of relevant trials has risen from a total of nine in 2009 to 180 by June 2020 worldwide (ClinicalTrials.gov), investigating the efficacy in treating various diseases such as type I and type II diabetes, liver cirrhosis, fistulas, cardiovascular disease, limb ischemia, amyotrophic lateral sclerosis, and lipodystrophy (reviewed in Ref. 20). While most trials are still in phase I/II, some early promising results have been published. ADSC injection has shown promise in treating Crohn's disease with no adverse effects but a higher healing rate and a lower recurrence rate.^21,22^ In acute myocardial infarction, transplantation of ADSCs significantly reduced the infraction size and perfusion defect in phase I/II clinical trials.^23^ Furthermore, ADSCs are also investigated in clinical trials for a variety of musculoskeletal,^24,25^ immunological,^26,27^ pulmonary,^28,29^ and reconstructive^30,31^ applications. However, not all preclinical findings have translated into clinically meaningful improvements (reviewed in Ref. 32), highlighting a need to better define and refine the regenerative potential of ADSCs.

The mechanism of action of ADSCs (and MSCs in general) on tissue regeneration is not well characterized. In fact, substantial argument still exists regarding what these cells actually are (stem cells vs stromal cells vs pericytes vs connective tissue cells vs a heterogeneous combination).^33,34^ Based on the multipotency of MSCs *in vitro* and their role in maintaining tissue homeostasis *in vivo*, it is natural to conclude that they contribute to tissue regeneration by differentiating into target cells. However, few reports demonstrate direct contribution of injected MSCs to injured tissue, and they are now thought to elicit therapeutic effects primarily through paracrine signaling and immunomodulation (reviewed in Ref. 35). This is true of ADSCs as well, both in a therapeutic context and in their native tissue environment (reviewed in Ref. 36). ADSCs may alter the tissue microenvironment through secretion of growth factors, inflammatory cytokines, or extracellular vesicles (exosomes) loaded with proteins, lipids, DNA mRNA, micro-RNA, tRNA, and noncoding RNA. Exosomes in particular have emerged recently as modulators of inflammation and tissue regeneration in the brain, heart, liver, kidney, and skin (reviewed in Ref. 37). If, as studies suggest, native ADSCs contribute significantly to the paracrine and endocrine action of adipose tissue^38,39^ and this action varies by depot, tailored to specific tissue targets, it is reasonable to suggest that (1) depot-specific ADSC sourcing could improve regenerative outcomes for specific cell-based therapies and (2) an improved understanding of depot-specific signaling could inform the appropriate sourcing and application of ADSCs. Tailoring ADSC sourcing to enhance the efficacy of translational therapies is not a new concept and has been the subject of several reviews;^40–42^ however, emerging data on ADSCs isolated from nontraditional depots suggest that another look beyond abdominal subcutaneous and visceral adipose tissue (VAT) is warranted.

## ADIPOSE TISSUE DIVERSITY IN FORM AND FUNCTION

### WAT, BAT, VAT, and SAT

Originally considered an inert storage depot for energy, our understanding of the role of adipose tissue has greatly expanded to now being considered the largest endocrine organ of the body.^43^ In addition to its role in energy balance, adipose tissue secretes a host of cytokines, termed “adipokines,” which regulate important biological processes and serve as markers for a multitude of cardiovascular, metabolic, and inflammatory diseases.^44^ Both the metabolic and endocrine functions of adipose tissue vary by depot.

Historically, adipose tissue is subdivided into two types: white adipose tissue (WAT) and brown adipose tissue (BAT). These two types are differentiated by gross appearance, cellular composition, gene expression signature, and developmental lineage (reviewed in Ref. 45). Specifically, BAT is brown in appearance, with mitochondria-dense, multilocular adipocytes that express uncoupling protein 1 (UCP-1) and derive from a myogenic factor 5 (Myf5) positive progenitor, while WAT appears white or yellow, with large UCP-1 and Myf5 negative unilocular adipocytes. They are also functionally distinct, with WAT storing energy in the form of triglyceride and BAT burning energy through adaptive thermogenesis. WAT is further divided into subcutaneous adipose tissue (SAT) and visceral adipose tissue (VAT). Unlike the classification of WAT vs BAT, which relies on fundamental differences within the adipose tissue, SAT and VAT are differentiated purely by anatomy, with SAT referring to all depots beneath the skin and VAT referring to all depots in the body cavity. Recent work has demonstrated considerable heterogeneity between depots within both the SAT and VAT categories^46,47^ and has even noted more similarity between some depots that cross categories than those within a category,^48^ calling into question the functional relevance of this anatomical classification.

Subcutaneous and visceral depots in the abdomen are the best characterized in humans. It was the subdivision of abdominal adiposity into these depots that launched the concept that not all WAT is created equal.^49^ Typically considered “good” fat, the deposition of abdominal SAT appears to have a cardio/metabolic-protective effect noted by increased antiatherogenic and antiinflammatory cytokines including interleukin 4 (IL-4) and transforming growth factor β (TGF-β). Opposingly, abdominal VAT as a “bad” fat is marked by heightened levels of lipolysis, immune cell infiltration, and proinflammatory adipokine release accompanied by metabolic detriments including higher risk of diabetes, dyslipidemia, and cardiovascular disease (reviewed in Ref. 50).

Recently, more attention has been paid to smaller VAT depots including fat around the vasculature, heart, and kidneys. Intriguingly, these depots have features of both BAT and WAT, where they resemble WAT in appearance but are capable of elevated UCP-1 expression and adaptive thermogenesis in response to β-adrenergic stimulation.^51–53^ This subtype of adipose tissue has been coined “beige,” “brite,” or “inducible brown” (reviewed in Ref. 45). It is believed that beige fat contains mature adipocytes that possess a bi-directional phenotype and fluctuate between a “dormant” state capable of storing energy like typical WAT and transition to an “active” state with the enhanced BAT function upon external stimulation. This distinctive phenotype in conjunction with the anatomical juxtaposition of these depots with specific tissues and organs suggests a unique function—likely distinct from classical abdominal SAT and VAT and potentially distinct from each other. The more that we uncover about adipose tissue, the more its inherent diversity defies the historical classification into WAT, BAT, VAT, and SAT. Thus, for the remainder of this review, individual depots will be named whenever possible.

### Adipose depots in rodents

**FIG. 1.**
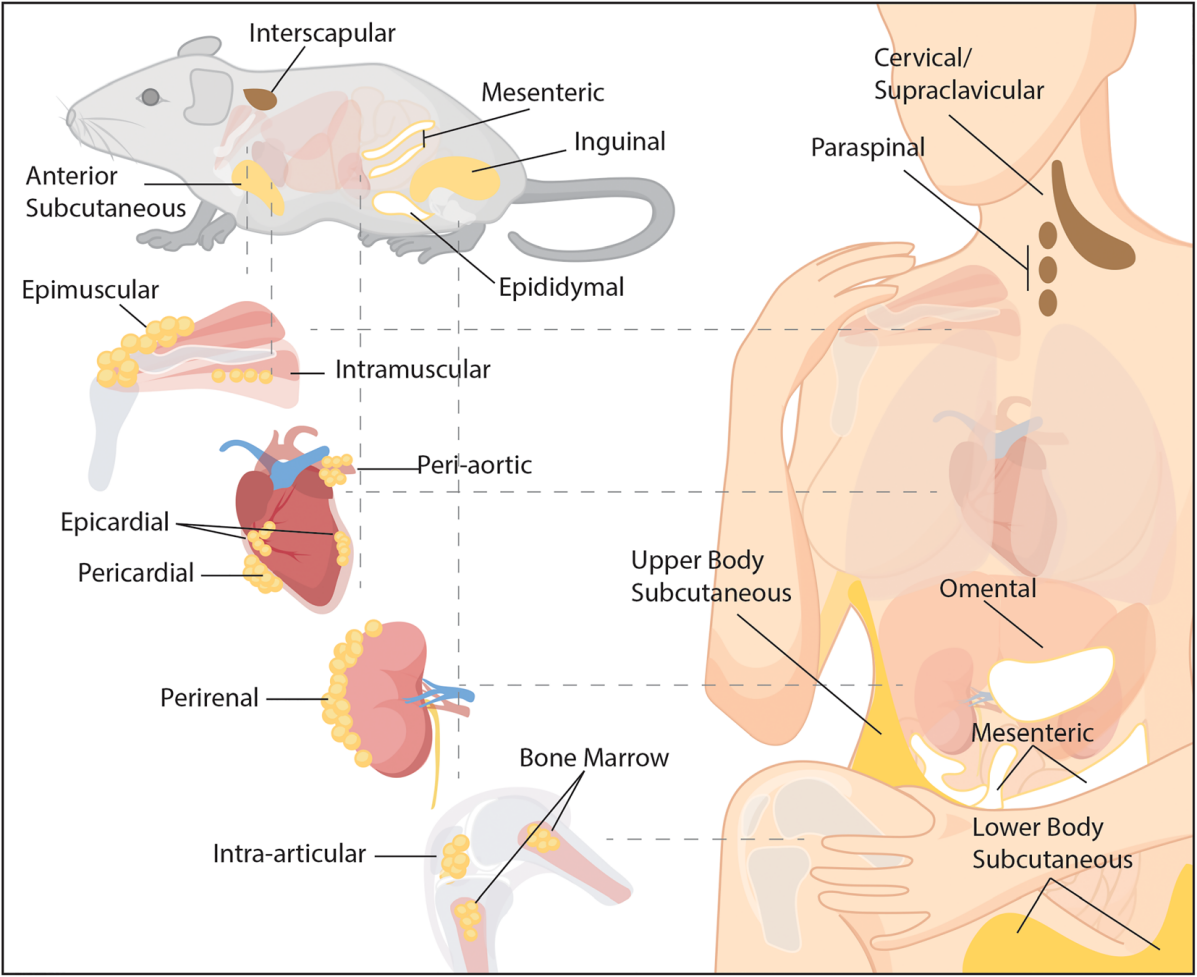
Anatomy of adipose depots in rodents and humans. The major brown (brown), beige (yellow), and white (white) depots are indicated in each species. Minor depots are depicted in callouts with the organ or tissue they are thought to support.

The most extensive characterization of adipose depots has been performed in mice and rats.^54–56^ Exact definitions vary per researcher, but the following fundamental anatomical locations seem to be the most agreed upon (Fig. 1). Murine SAT can be identified as four different depots: (1) inguinal fat is located subcutaneously posteriorly and runs dorsal along the hindlimb. It is the “classical” SAT and beige fat depot in rodents. (2) Anterior subcutaneous fat is a thin layer of WAT that runs on the sides of the ribcage. (3) Triceps-associated fat is a thin WAT depot located in the front legs and runs along the length of the triceps muscles. (4) Interscapular fat is located between the shoulder blades and is superior to the interscapular BAT fat pad. VAT can be identified as five depots in mice: (1) perigonadal/epididymal fat is considered prototypical visceral adipose in rodents and is located either attached to the epididymis and testis in males or near the ovaries in female mice. (2) Mesenteric fat lines the large intestines. (3) Perirenal fat is located posterior to the renal capsule of the kidneys. (4) Retroperitoneal fat is located anterior to the kidneys. (5) Cardiac fat is associated with the heart and can be further subdivided into epicardial adipose tissue, which is located between the myocardium and the visceral pericardium and pericardial fat that lies between the visceral and parietal pericardium. Classical BAT in rodents can be considered SAT or VAT depending on the anatomical location. Typically, it is defined as the four following depots: (1) Interscapular fat is considered the classical BAT depot and is present in two lobes between the scapulae. (2) Cervical fat is two lobe-shaped fat pads located beneath the scapulae and head. (3) Perirenal fat in its brown form is distinct from perirenal VAT and is located on the hilum side of the kidney. (4) Axillary fat is located anterior to the anterior subcutaneous WAT. In addition to these, adipose tissue can be identified surrounding blood vessels (perivascular fat, reviewed in Ref. 57), within synovial joints (articular fat pads, reviewed in Ref. 58), around and within muscle (epimuscular and intramuscular adipose tissue, reviewed in Ref. 59), and within bone marrow (bone marrow adipose tissue, reviewed in Ref. 60). Rather than existing as a single depot, this “auxiliary” adipose is composed of discrete small depots throughout the body. Although the small depots are frequently grouped under a single heading (e.g., perivascular), evidence suggests that physiology and phenotype vary with the anatomical location (e.g., peri-thoracic aortic vs peri-abdominal aortic).^61–63^

Comparative studies between depots demonstrate unique paracrine and endocrine functions, as well as marked differences in their contribution to health. As discussed above, by far, the most comparative data exist between the classical SAT and VAT depots. In mice, these are inguinal and epididymal fat. Extensive transcriptomic, proteomic, and secretomic studies reveal differences in the metabolic function and adipokine profiles both at the baseline^39,64,65^ and in response to stimuli (e.g., high fat feeding or exercise).^66,67^ However, perhaps the most convincing evidence for intrinsic differences in these depots comes from fat transplant studies. Stanford *et al.* transplanted inguinal and epididymal adipose from sedentary and exercise-trained mice into the subcutaneous space or the visceral cavity.^68^ Transplanting inguinal fat from exercised mice to either location of sedentary mice improved glucose tolerance. However, no improvements were found when transplanting exercised epididymal fat, suggesting that the source of the transplant, rather than its anatomical location, defines its physiological contribution.

In addition to the heterogeneous endocrine contributions of the major SAT and VAT depots, there appear to be specialized paracrine roles for many of the smaller auxiliary adipose depots. For example, the close proximity of perivascular adipose tissue and the blood vessel enables secreted adipokines to act locally either through the vasa vasorum or directly through the vessel wall (reviewed in Ref. 57). Indeed, mechanistic studies in rodents have demonstrated unique action of perivascular adipokines on vascular tone^69^ and proliferation of smooth muscle cells.^70^ Similarly, paracrine adipokines are proposed to underlie the cardioprotective effects of epicardial fat (reviewed in Ref. 71) and the cartilage protective effects of the infrapatellar fat pad (IPFP) (reviewed in Ref. 72). However, the vast majority of the literature concerning paracrine actions of these depots covers their negative role in disease states. Taken together, it suggests that paracrine adipose signaling, like endocrine adipose signaling, is plastic—promoting homeostasis in health and pathology in disease.

### Adipose depots in humans

Similar to rodents, adipose tissue in humans is a multidepot organ; however, specifics of the depots differ (Fig. 1). In humans, abdominal VAT is classically subdivided into omental (anterior to the intestines), mesenteric (surrounding the intestines), and retroperitoneal (posterior to the abdominal organs) depots.^73^ Rodents mainly store VAT in the perigonadal depot, with the most analogous VAT in humans being mesenteric fat.^54^ On the flip side, while the omental VAT depot can be expansive in humans, it is not easily identifiable in mice. These differences do not allow for clear parallels between VAT in species. Human SAT is typically subdivided into upper-body and lower-body regions.^73^ Unlike in rodents where it exists in discrete depots (e.g., inguinal), in humans, SAT is distributed throughout the body, though the thickness of the fat varies by region and preferential storage varies by individual.

Defining BAT depots in humans has been complicated by the fact that adipose in anatomical locations corresponding to rodent BAT more resembles rodent beige fat than classical BAT.^74^ Indeed, in humans, most of the classical BAT is thought to be lost in infancy and replaced with a beige spectrum of inducible thermogenesis.^75,76^ In addition to the typical paraspinal and supraclavicular depots, most of the auxiliary organ- and tissue-associated depots exhibit a beige signature. What data are available suggests functional overlap with rodent depots as well. Peri-thoracic aortic adipose tissue expresses transcriptional markers of beige/brown fat in mice^77^ and humans.^52^ The vasodilatory action of this fat has been confirmed in human explants^78^ and appears to be lost in the context of obesity.^79^ Similarly, epicardial and rotator cuff epimuscular adipose tissues have confirmed beige signatures in humans^51,80^ and in mice^54^ (our unpublished data). Epicardial adipose secretion of a number of adipokines identified in rodent studies, including adiponectin, adrenomedullin, and omentin-1, is confirmed to be correlated with the cardiac function in humans.^81–83^ Coculture models of human epimuscular adipose–muscle crosstalk also suggest that paracrine adipokines could regulate myogenesis.^80^ Interestingly, epicardial and epimuscular fat exhibit a concurrent decline in these “protective” effects and a transcriptional shift toward white fat in the presence of coronary artery disease and chronic rotator cuff disease, respectively,^80,84^ suggesting that phenotypic transitions could cause these fats to contribute negatively to disease states. Similarly, perirenal adipose tissue has a brown or beige phenotype in both humans^53,85^ and rodents,^54^ with a specific classification that varies by location and study. While the physiological role of perirenal adipose remains uncharacterized, it is responsive to obesity and type 2 diabetes where its expansion is linked to renal dysfunction in both species.^86,87^ Evidence in mice and humans suggests that bone marrow adipose tissue does not fully resemble brown, beige, or white fat and may have a unique phenotype.^62,88^ Paracrine actions of this adipose depot on bone remodeling are proposed in both mice and humans, but strong mechanistic supporting data are lacking in both species (reviewed in Ref. 89). Finally, while the infrapatellar fat pad is thought to be a white phenotype in both rodents and humans, it is also thought to be unique in its metabolic regulation.^90,91^ It has long been thought to be involved in the progression of osteoarthritis, likely through paracrine inflammatory adipokines (reviewed in Ref. 92) but intriguingly, ADSCs isolated from this depot have also been shown to ameliorate the condition (reviewed in Ref. 72).

Amassed together, this substantial and growing body of evidence suggests that each adipose depot is unique in form and function. Most relevant to this review are notable differences in paracrine and endocrine secreted adipokines, which are likely to arise in part from the ADSC compartment.^38,39^ If ADSCs from different depots are tailored to uniquely regulate specific tissue functions and they maintain this purpose upon isolation, then selection of the source of ADSCs should be similarly tailored to maximize regenerative potential.

## ADSC REGENERATIVE POTENTIAL ACROSS DEPOTS

Given the diversity in adipose tissue, it is not surprising that the characteristics of the ADSC population also vary by depot. However, before a comparative discussion can be undertaken, it must be noted that even within a single depot, the ADSC population is heterogeneous (reviewed in Ref. 93) with 2–3 distinct subpopulations noted in mouse epididymal and inguinal depots. Therefore, differences between depots at the population level could arise from intrinsic differences in each ADSC, differences in specific subpopulations, or differences in the fractional composition of the depot. Only studies using single cell or clonal analyses can differentiate between these possibilities. Further complicating the generalization of ADSC characteristics, recent data indicate that lineage and transcriptional markers may identify different subpopulations in different depots.^54,94^ For instance, while the ADSCs that give rise to thermogenic adipocytes in BAT hail from a Myf-5 positive lineage,^95^ Myf-5 positive ADSCs in SAT are not the source of thermogenic beige adipocytes.^94^ Taken together, these data point to the need to individually characterize subpopulations in each depot to fully realize the translational potential of ADSCs. While most current translational work remains focused on the collective ADSC population of abdominal SAT, future identification of subpopulations with unique surface markers and regenerative potentials could hone the use of even the standard adipose source material, optimizing ADSC selection, and improving outcomes. However, as our understanding of inter- and intra-depot ADSC heterogeneity is still in its infancy, most comparative data exist at the population level.

In line with the characterization of adipose tissues, the most extensive comparison of ADSCs is between those derived from the classical SAT and VAT depots (inguinal and epididymal in mice and abdominal subcutaneous and omental in humans). Generally speaking, they have significant similarities: they both are stable in culture, exhibit similar degrees of pluripotency, and share a surface marker profile.^96,97^ However, a deeper comparison reveals subtle but significant differences (reviewed in detail in Ref. 42). Numerous studies in mice and humans confirm higher *in vitro* proliferation and adipogenic and chondrogenic differentiation rates in ADSCs derived from SAT compared with VAT,^96,97^ while VAT ADSCs have higher osteogenic potential.^98^ Proliferative differences are supported by microarray analysis, indicating that VAT ADSCs have upregulated clusters of genes related to lipid synthesis and metabolism, while SAT ADSCs have highly expressed genes in DNA-dependent transcription.^96^ Ease of access and expansion certainly make SAT ADSCs an appealing therapeutic choice, and indeed, abdominal SAT is the primary source for clinical and preclinical ADSC based regenerative studies—but lineage preference during directed differentiation suggests that intrinsic differences might control the efficacy per quantity of transplanted cells. Secretome analyses support this hypothesis as, compared with abdominal SAT ADSCs, omental VAT ADSCs secrete elevated levels of inflammatory cytokines (IL-6 and IL-8)^99,100^ thought to be involved in immunomodulation of wound healing.^101^ However, the few studies that have investigated this directly have found both sources to equally promote cardiac and dermal regeneration in rodents.^102,103^

ADSCs can also be derived from BAT in humans and rodents and exhibit similar characteristics in culture to their WAT counterparts: fibroblastic morphology, stable expansion, multipotency, and mesenchymal surface marker expression,^104–106^ although the degree of potency and level of expression may differ.^107^ One notable exception is that BAT-derived ADSCs differentiate into adipocytes with high expression of thermogenic regulatory genes (Ucp1, Cidea, and Pgc1α) and elevated uncoupled respiration^76,105^ compared with those derived from WAT. This suggests that the ADSCs retain a differentiation memory from their tissue of origin: BAT-derived and WAT-derived ADSCs want to differentiate into brown and white adipocytes, respectively, in the same culture conditions. A second notable exception is that BAT-derived ADSCs exhibit elevated potential for differentiation down the cardiomyocyte lineage,^106,108^ which is currently being explored for its therapeutic potential. Aside from these studies, the vast majority of BAT ADSC-based work has focused on delineating factors that control the browning response or have used differentiated adipocytes as a model system for BAT. Comparisons of the transcriptional or secretory profiles between undifferentiated BAT and WAT ADSCs are lacking. However, adipocytes derived from BAT ADSCs maintain secretion of some BAT associated adipokines, and their secretome is responsive to adrenergic (browning) stimulation *in vitro*.^109^ Specifically, simulation with norepinephrine increases secretion of adipokines with antiinflammatory action in interscapular BAT ADSC-derived adipocytes but not in those derived from inguinal WAT.^109^ This suggests that even in isolation, the BAT ADSC secretome retains a unique responsiveness to environmental cues, which could be harnessed for regenerative applications.

A handful of studies have explored the unique features and regenerative potential of ADSCs isolated from depots outside the classical WAT and BAT described above (Table I). The most extensive characterization has been in the infrapatellar fat pad (IPFP)—an intraarticular fat pad that sits in the knee joint behind and just below the patella. Investigations in this small adipose depot were initially driven by studies suggesting that it played a role in joint pathologies such as post-arthroscopy fibrosis. As it is easily accessible during knee surgeries (and frequently removed as surgical waste), it has been well characterized *in vitro*. Generally, ADSCs isolated from the IPFP resemble those isolated from SAT in the surface marker profile, proliferative capacity, and potency—though reports vary as to which source exhibits higher proliferation and osteogenic capacity.^110,111^ However, there is a consensus that IPFP derived ADSCs have increased chondrogenic potential compared with other adipose-derived stromal cell (ADSC) sources, with numerous studies showing increased chondrogenic gene expression and production of cartilage matrix in directed differentiation.^110–113^ This finding combined with promising preclinical and clinical outcomes treating osteoarthritis with marrow derived MSCs and SAT derived ADSCs suggests that IPFP derived ADSCs could represent a unique therapeutic cell source. While no studies have yet pitted IPFP and SAT derived ADSCs against each other, injection of IPFP ADSCs into osteoarthritic animal models and human knees has improved radiological and functional outcomes, indicating that they can indeed impact degeneration.^114–116^

**TABLE I.** Preclinical and clinical studies utilizing ADSCs from nontraditional depots for regenerative medicine applications.

Source	References	Applications	Outcomes
Infrapatellar fat pad	114	Preclinical osteoarthritis model (rabbit); intraarticular ADSC injection	Improved cartilage quality, reduced subchondral sclerosis compared wilt cell-free media
	154	Preclinical osteoarthritis model (mouse); intraarticular exosome injection	Improved cartilage quality, enhanced matrix synthesis compared with exosome-free media
	116	Therapeutic case-control study, osteoarthritis; intraarticular ADSC injection with platelet rich plasma	No significant change in activity or pain scores compared with cell-free media
	115	Therapeutic case-control study, osteoarthritis; intraarticular ADSC injection with platelet rich plasma	Improved activity and pain scores correlated with the number of injected cells
Pericardial adipose tissue	153	Preclinical myocardial infarction model (rat); infarct border zone ADSC injection	Increased ventricular wall thickness and ejection fraction in Wilms' tumor factor 1 positive ADSCs compared with negative
	117	Preclinical myocardial infarction model (mouse and rat); ADSC injection	Reduced infarct size and ejection fraction compared with cell-free media
	119	Preclinical myocardial infarction model (rat); infarct center ADSC injection	Increased ventricular wall thickness and ejection fraction compared with inguinal derived ADSCs
	121	Preclinical myocardial infarction model (rat); infarct center ADSC injection	Increased ventricular wall thickness and ejection fraction in flk-1 positive ADSCs compared with negative
Perirenal adipose tissue	130	Preclinical pyelonephritis model (rabbit); subscapular ADSC injection	Improved interstitial fibrosis and cortical function compared with inguinal derived ADSCs
	131	Preclinical ischemia-reperfusion liver injury model (mouse); delivery method not specified	Reduced cellular necrosis and increased survival compared with untreated control
	132	Preclinical muscle regeneration model (rabbit); intramuscular ADSC injection	Increased muscle mass and contractile force compared with cell-free media

ADSCs derived from the cardiac-associated depots—epicardial and pericardial fat—have also been examined for their potential in cardiac regeneration. Epicardial fat is adjacent to the myocardium without a fascial division, while pericardial fat lies on the outer surface of the fibrous pericardium. ADSCs from both depots exhibit a typical mesenchymal morphology, surface marker profile, and potency,^98,117,118^ but, like BAT-derived ADSCs, they exhibit increased cardiomyocyte differentiation potential compared with abdominal SAT and VAT in both humans and rodents.^117–119^ A similar propensity for cardiomyocyte differentiation is found in perivascular fat around the aorta, which is also sometimes classified as cardiac adipose.^117,120^ In animal models of cardiac ischemia, injection of pericardial ADSCs increased the thickness of the infarcted wall with improved vascularization and function compared with injection of inguinal ADSCs,^117,119,121^ suggesting that cardiac ADSCs could elicit improved regenerative responses in the heart. Interestingly, despite their cardiomyogenic potential, the cells did not appear to contribute directly to the regenerating tissue but rather were thought to improve vascularization and myogenesis via secreted paracrine factors.^117,119^

A similar but less characterized depot of fat can also be found around some skeletal muscles—most notably the muscles of the rotator cuff. Rotator cuff epimuscular fat sits on the superior surface of the supraspinatus muscle, outside the fascial boundary of the muscle. Human ADSCs derived from this fat exhibit increased myogenic potency *in vitro* compared with subject-matched SAT ADSCs and are able to directly contribute to regenerating muscle *in vivo*.^80^ Interestingly, the progenitor cells that give rise to the other skeletal muscle associated adipose, intramuscular fat (adipocytes between myofibers within the fascial boundary of the muscle) do not exhibit myogenic potency either *in vitro* or *in vivo*,^122,123^ highlighting the distinct characteristics of these two fats. Despite lacking myogenic potency, these muscle-resident fibro/adipogenic progenitor (FAP) cells are thought to facilitate regeneration, likely through paracrine signaling.^122,123^ While FAPs are less abundant and more difficult to isolate than ADSCs derived from traditional adipose depots, they are still being explored as a potential source for cell-based regenerative therapies in muscles.^124^

ADSCs derived from perirenal adipose tissue likewise exhibit an MSC-associated surface marker profile and are capable of trilineage differentiation in rodents and humans.^125–127^ Additionally, studies have demonstrated immunomodulation by these cells in both species.^126,128^ The degree of similarity between perirenal ADSCs and those from classical VAT and SAT depots depends on which portion of the fat is investigated.^53^ In rodents, perirenal fat is composed of a WAT and a BAT layer and in humans exhibits a graded regional variation in brown features, with the most prominent being close to the hilus.^85^ ADSCs derived from perirenal WAT are transcriptionally very similar to those derived from epididymal WAT in rats,^129^ while perirenal BAT ADSCs resemble human perithyroid BAT and mouse interscapular BAT.^85^ Only a few studies have compared ADSC differentiation potentials between perirenal and other adipose sources. To date, the only noted differences are higher proliferation,^47^ adipogenic potential,^47^ and capacity to differentiate into Schwann-like cells^127^ compared with epididymal ADSCs. Despite the lack of *in vitro* characterization, perirenal ADSCs have been applied therapeutically in preclinical models of disease with promising results. In a rabbit model of acute pyelonephritis, injection of autologous perirenal ADSCs enhanced histopathological outcomes and significantly improved the cortical function compared with neck subcutaneous ADSCs.^130^ Additionally, systemic injection of perirenal ADSCs improved liver regeneration in a mouse model of ischemia-reperfusion injury^131^ and muscle regeneration in a rabbit model of toxin-induced injury.^132^ However, no comparison was made between ADSC sources in these studies.

The progenitor cells that give rise to the adipocytes in the bone marrow are a unique subpopulation within the MSCs derived from the bone marrow.^133,134^ They are closely associated with the bone marrow adipocytes^135^ and, in their niche, support neo-vascularization and bone regeneration through adipokine secretion.^133,134^ While bone marrow derived MSCs have been extensively characterized *in vitro* and evaluated in regenerative therapies *in vivo*, the contribution of this specific subpopulation has yet to be defined. Identification of unique surface markers could enable purification of these cells to harness their proregenerative paracrine signaling for regenerative therapies.

While extensive characterization is lacking and there may be a literature bias toward pairing ADSCs derived from specific auxiliary depots with regenerative therapies targeting the associated organ/tissue, what evidence exists suggests that selective ADSC sourcing could improve regenerative medicine strategies. Importantly, not only does there appear to be a propensity for ADSCs to support regeneration in the organ/tissue with which they are associated, but also these ADSCs are frequently accessible during the surgical treatment of that organ/tissue. For example, the IPFP is accessible during knee osteotomy, pericardial fat is accessible during open heart surgery, and epimuscular fat is accessible during rotator cuff repair. In each of these scenarios, surgical success will depend on the degree of tissue regeneration and healing over subsequent months, and thus, post-surgical adjuvant treatment with ADSCs isolated during the surgery is an intuitive and feasible approach to improve outcomes. However, such strategies will face biological and technical challenges. Most significant among them is the limited source material. In comparison to abdominal SAT, these auxiliary depots are very small, which will likely necessitate culture expansion of ADSCs to generate sufficient numbers to achieve therapeutic benefits. Second, as mentioned previously, most of these depots undergo pathologic changes in response to disease in their associated organ/tissue and may even contribute to the organ/tissue pathology. It may not be beneficial to directly apply ADSCs isolated from the diseased fat to the diseased organ. However, part of the therapeutic potential of ADSCs lies in their plasticity and ability to be redirected by environmental cues. Significant advances have been made in tissue engineering to recreate healthy environmental cues *in vitro* and *in vivo* to maximize survival and senescence-free expansion and direct differentiation and adipokine secretion to maximize the pro-regenerative impact of stem cell-based therapies. Harnessing these strategies could make any adipose depot a realistic source for therapeutic ADSCs.

## NOVEL STRATEGIES TO OPTIMIZE ADSC-BASED THERAPEUTICS

The field of tissue engineering has been working for over a decade to optimize ADSC-based therapeutics. The breadth of innovative strategies and designs has filled several reviews,^136,137^ but generally speaking and for the purpose of a brief introduction, engineering approaches can be broken down into four main categories: scaffold design, cytokine delivery, genetic engineering, and other culture environment modifications. Scaffold engineering takes advantage of the sensitivity of ADSCs to the stiffness, topography, and composition of the extracellular matrix (ECM). Scaffolds can be employed *in vitro* to promote quiescence or senescence-free expansion or to direct differentiation prior to *in vivo* applications. Alternatively, they can be applied with ADSCs to promote survival, reduce dispersion, and direct differentiation *in vivo*. Cytokine delivery approaches seek to direct ADSC behavior using soluble factors in the microenvironment and are frequently used in combination with engineered scaffolds to combine physical and chemical cues. Most of the current strategies either deliver growth factors to promote cell survival or deliver inflammatory cytokines to enhance the immunomodulatory effects of ADSCs. Genetic engineering can be used to elicit a stronger and more sustained survival and directed differentiation response by editing apoptotic genes and the transcription factors that control pluripotency. Finally, culture conditions are continually being optimized to improve ADSC survival and performance in diseased microenvironments. Two major strategies are hypoxic preconditioning and 3D spheroid culture. Both strategies are designed to better mimic the ADSC native microenvironment and have been shown to improve the viability and survival of transplanted ADSCs.

**FIG. 2.**
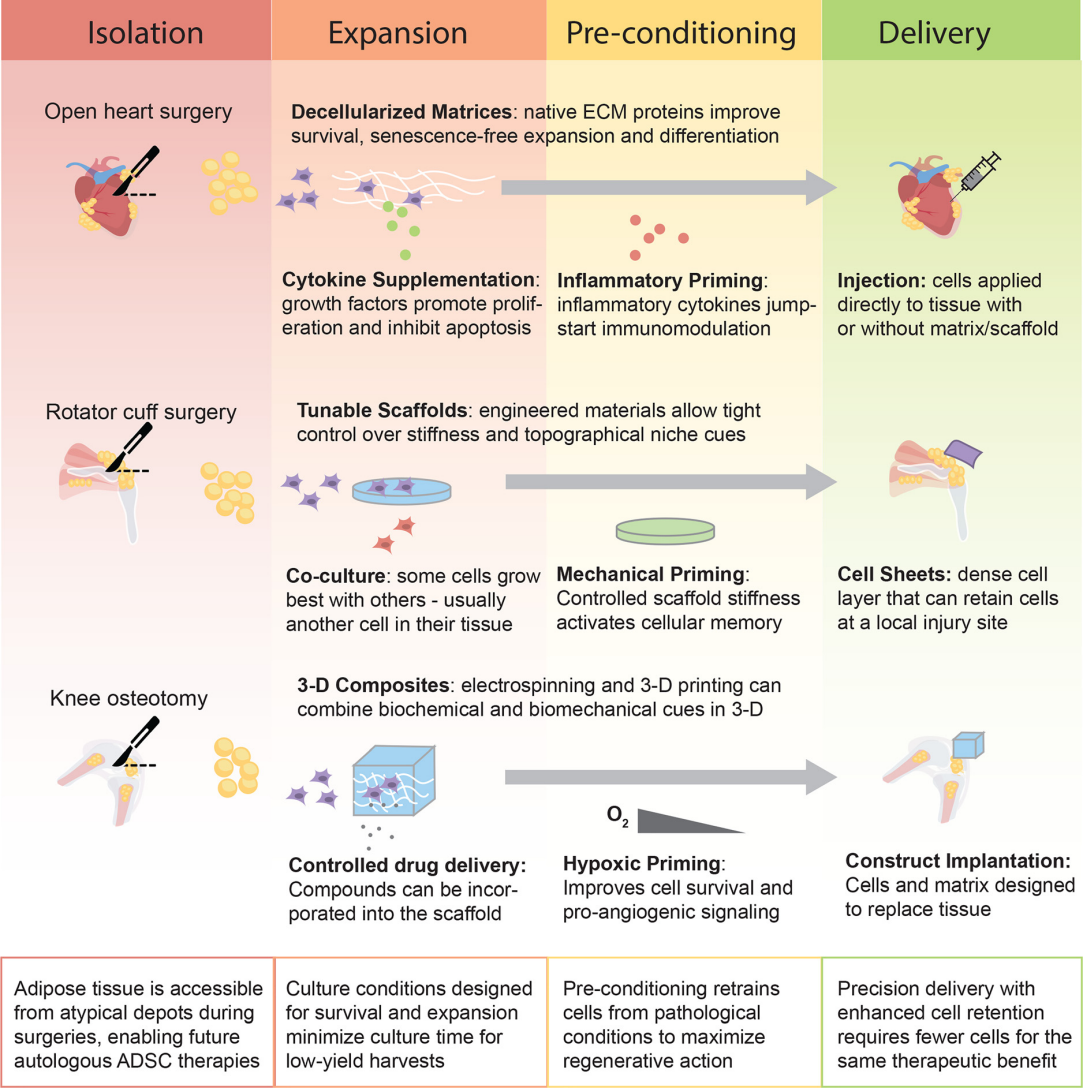
Engineering strategies to enhance the use of ADSCs from nontraditional depots in regenerative medicine. These strategies may be applied in isolation or in combination to any number of depots.

These strategies were developed and optimized with ADSCs derived from abdominal SAT, but they have clear potential for improving the translational potential of ADSCs derived from other depots (Fig. 2). The limited quantity of source material limits the number of ADSCs that can be isolated from smaller adipose depots. Expansion of the population in culture can overcome this issue, but as ADSCs are highly sensitive to microenvironmental cues and retain an environmental memory,^138^ longer culture times outside the native microenvironment could result in poorer outcomes. Engineering strategies mimicking microenvironmental cues in culture have significantly improved expansion rates, survival, and potency in SAT derived ADSCs (reviewed in Ref. 136). Similarly, when native ECM proteins were used as a culture surface (rather than tissue culture plastic), proliferation and chondrogenic and adipogenic potency were enhanced in IPFP ADSCs.^139^ Proliferation of perirenal ADSCs was also increased by culture on the decellularized ECM.^140^ While these coatings provide some of the native cell–protein interactions, they lack the native topography. 3D printing and electrospinning of ECM proteins can combine structural and biochemical cues to further guide cellular behavior. *In vitro*, induced osteogenic differentiation of epicardial ADSCs is improved with culture on electrospun collagen.^141^ Electrospun collagen matrices^142^ and bioprinted ECM^143^ have also been used to develop tissue engineered constructs from IPFP ADSCs. These constructs are designed for implantation to improve the survival and efficacy of transplanted cells *in vivo*, thereby requiring fewer ADSCs to elicit a therapeutic effect.

Chronic diseases such as aging and diabetes impair both the potency and immunomodulatory functions of SAT ADSCs.^144,145^ Given the pathological changes to many of the tissue/organ associated fat depots in the conditions in which they would be accessible (e.g., major surgery), disease and aging are likely to affect the translational potential of autologous ADSCs from these depots as well. Indeed, obesity impairs the chondrogenic potential of IPFP ADSCs^146^ as likely does osteoarthritis.^147^

However, IPFP ADSCs isolated from arthritic knees can still be conditioned to generate tissue engineered cartilaginous grafts *in vitro* using a combination of hypoxic preconditioning, biochemical cues, and biophysical cues.^148,149^ Similarly, the angiogenic function of epicardial ADSCs isolated from diabetic rats can be rescued by pretreatment with a soluble Notch pathway inhibitor.^150^ While deficits have not been investigated in perirenal ADSCs in the context of aging or metabolic derangement, inflammatory priming has been shown to enhance their immunosuppressive capacity.^151^ These strategies to “recondition” ADSCs isolated from aged tissue or disease states (hypoxic and inflammatory priming in particular) have been very effective in restoring the regenerative potential of SAT derived ADSCs (reviewed in Ref. 152). Interestingly, pericardial ADSCs were shown to respond to myocardial infarction by switching to a pro-repair phenotype,^153^ suggesting that sourcing ADSCs from some injury/disease states could confer a regenerative benefit. In this study, Tang *et al.* were able to identify a surface marker to sort the proregenerative ADSCs to maximize the therapeutic benefit of cell injection.^153^ Alternatively, it is possible to isolate ADSCs from these depots in healthy adults in situations of organ and tissue donation. In this case, cell-free strategies harnessing paracrine signals present in exosomes could overcome both the issue of autologous ADSC dysfunction and allogenic ADSC rejection.^154^ Exosomes derived from human IPFP ADSCs were recently shown to protect articular cartilage in a mouse model of osteoarthritis, demonstrating that the regenerative power of exosomes can be harnessed outside of traditional abdominal adipose depots.^154^

ADSC based therapies sourcing from these nontraditional depots may also be able to capitalize on some new engineering strategies targeting beige and brown fat. While studies have yet to fully differentiate the regenerative potential—and heterogeneity—of ADSCs derived from the nonwhite fats, many have hypothesized that their paracrine effects are modifiable by adrenergic (browning) stimuli. Positive selection of progenitors that are sensitive to browning stimuli, priming with browning stimuli prior to cell delivery and delivery of cells in conjunction with browning stimuli all have the potential to further direct the regenerative action of beige fat derived ADSCs. While current engineering strategies are focused on generating and transplanting mature BAT,^155,156^ they have demonstrated the value of optimizing engineering strategies to brown and beige progenitors.

As our understanding of the diversity of ADSCs expands, population-specific engineering will likely play a significant role in expanding the use of ADSCs in regenerative medicine. However, a word of caution is warranted as much remains to be done at the preclinical and clinical levels before ADSCs can be effectively used in therapeutically. A heterogeneous stromal vascular fraction (SVF) containing ADSCs has become a popular therapy, sometimes discussed as a “cure-all” despite a lack of careful characterization and consistent documentation of clinical efficacy. Variability in outcomes across clinical trials, and in clinics, no doubt reflects a lack of uniformity in isolation and delivery, as well as a limited understanding of the factors that influence the transplanted ADSC behavior. A step back to fundamentally understand these cells and their heterogeneity will enable guided, targeted ADSC-based therapeutics with consistent and positive outcomes.

## CONCLUSIONS

There is incredible diversity in function across adipose depots, and data support the concept that each depot is specialized to play a specific role in homeostasis and disease. At least some of this specialization is retained in isolated ADSCs. Preferential potencies, different cytokine secretion profiles, and varied immunomodulatory capacities suggest that ADSCs from specific sources may be optimal for specific applications. While much more characterization and comparative data are needed, work to date strongly suggests that improving methodologies to utilize ADSCs from multiple depots could significantly improve regenerative medicine outcomes.

## AUTHORS' CONTRIBUTIONS

C.G. and J.P. contributed equally to this work.

## Data Availability

Data sharing is not applicable to this article as no new data were created or analyzed in this study.
